# Priority index for critical Covid-19 identifies clinically actionable targets and drugs

**DOI:** 10.1038/s42003-024-05897-0

**Published:** 2024-02-16

**Authors:** Zhiqiang Zhang, Shan Wang, Lulu Jiang, Jianwen Wei, Chang Lu, Shengli Li, Yizhu Diao, Zhongcheng Fang, Shuo He, Tingting Tan, Yisheng Yang, Kexin Zou, Jiantao Shi, James Lin, Liye Chen, Chaohui Bao, Jian Fei, Hai Fang

**Affiliations:** 1 Shanghai Institute of Hematology, State Key Laboratory of Medical Genomics, National Research Center for Translational Medicine at Shanghai, Ruijin Hospital, Shanghai Jiao Tong University School of Medicine, Shanghai, 200025 China; 2 School of Life Sciences and Biotechnology, Shanghai Jiao Tong University, Shanghai, 200240 China; 3 Translational Health Sciences, University of Bristol, Bristol, BS1 3NY UK; 4 Network and Information Center, Shanghai Jiao Tong University, Shanghai, 200240 China; 5 MRC London Institute of Medical Sciences, Imperial College London, London, W12 0HS UK; 6 Precision Research Center for Refractory Diseases, Institute for Clinical Research, Shanghai General Hospital, Shanghai Jiao Tong University School of Medicine, Shanghai, 201620 China; 7 College of Finance and Statistics, Hunan University, Changsha, 410079 Hunan China; 8 College of Health Science and Technology, Shanghai Jiao Tong University School of Medicine, Shanghai, 200025 China; 9 Key Laboratory of RNA Science and Engineering, Shanghai Institute of Biochemistry and Cell Biology, Center for Excellence in Molecular Cell Science, Chinese Academy of Sciences, Shanghai, 200031 China; 10 Nuffield Department of Orthopaedics, Rheumatology and Musculoskeletal Sciences, University of Oxford, Oxford, OX3 7LD UK; 11 Department of General Surgery, Ruijin Hospital Luwan Branch, Shanghai Jiao Tong University School of Medicine, Shanghai, 200020 China; 12 Department of General Surgery, Pancreatic Disease Center, Ruijin Hospital, Shanghai Jiao Tong University School of Medicine, Shanghai, 200025 China

**Keywords:** Software, Infectious diseases, Target identification

## Abstract

While genome-wide studies have identified genomic loci in hosts associated with life-threatening Covid-19 (critical Covid-19), the challenge of resolving these loci hinders further identification of clinically actionable targets and drugs. Building upon our previous success, we here present a priority index solution designed to address this challenge, generating the target and drug resource that consists of two indexes: the target index and the drug index. The primary purpose of the target index is to identify clinically actionable targets by prioritising genes associated with Covid-19. We illustrate the validity of the target index by demonstrating its ability to identify pre-existing Covid-19 phase-III drug targets, with the majority of these targets being found at the leading prioritisation (leading targets). These leading targets have their evolutionary origins in Amniota (‘four-leg vertebrates’) and are predominantly involved in cytokine-cytokine receptor interactions and JAK-STAT signaling. The drug index highlights opportunities for repurposing clinically approved JAK-STAT inhibitors, either individually or in combination. This proposed strategic focus on the JAK-STAT pathway is supported by the active pursuit of therapeutic agents targeting this pathway in ongoing phase-II/III clinical trials for Covid-19.

## Abstract (web summary)

A priority index solution to critical Covid-19 comprises both the target index and the drug index, serving as dual indexes to accelerate translational medicine for host genetics.

## Introduction

Host genetics, encompassing human genetic contributions to infectious diseases, holds the potential to unveil genetically informed mechanisms for disease prevention and drug therapy. Naturally occurring genetic variations within the host genome have catalysed strategies in preventing infectious diseases, often referred to as ‘natural immunity’. Successful examples include the discovery of HIV-1 infection natural resistance attributed to the *CCR5* gene^1^, the identification of the malaria resistance locus at the *HBB* gene^2^, and the protective effects conferred by the *WT1* locus against tuberculosis^3^. In addition to providing evidence of disease prevention mechanisms, these host variations can offer insights into therapeutic targeting by mimicking the on-target effects of pharmacological interventions, known as ‘clinical trials by nature’^4^. Moreover, genetic variations can exert regulatory influence on genes (‘effector genes’), which encode protein targets of drugs — both those approved/licensed and those in clinical phase development — thus providing opportunities for repurposing pre-existing drugs into new indications^5,6^.

Genetic targets are defined as early-stage genetically informed and validated therapeutic candidates^7^. The endorsement of genetic targets enhances the chance of approval along the drug development pipeline, as compared to drug-target pairs without such genetic backing^8,9^. The proposition of genetic targets is timely, particularly amid the pandemic crisis brought about by coronavirus disease 2019 (Covid-19), attributed to the severe acute respiratory syndrome coronavirus 2 (SARS-CoV-2)^10^. Its severity exhibits strong correlations with factors such as older age, being male, lower socio-economic status, non-European demographic ancestry, and pre-existing clinical comorbidities (including diabetes)^11^. Nonetheless, these non-genetic factors do not fully explain disease severity, as instances of severe cases among young, otherwise healthy individuals, often run in families^12^ and persistently hint at genetic contributions to disease severity^13^.

In a fast-moving landscape of Covid-19 field^14,15^, there emerges a need for the timed translation of host genetic findings into precise therapeutics targeted to treat critically ill patients with life-threatening Covid-19 (referred to herein as ‘critical Covid-19’). Individuals suffering from critical Covid-19 are likely to develop acute respiratory distress syndrome, accompanied by a hyperinflammation phenotype characterised by the release of excessive pro-inflammatory cytokines, resembling immune-related disorder manifestations^16^. With the continual expansion of sample sizes and a clearer definition of disease severity, genome-wide studies^17–24^ offer a wealth of genetic targets pertinent to critical Covid-19. Nonetheless, the task of identifying genetic targets is intricate, primarily due to the prevalence of disease severity loci within the non-coding human genome. This challenge is further exacerbated by the regulatory nature of non-coding loci on effector genes, often involving long-range and cell-type-specific mechanisms^25^.

**Fig. 1:**
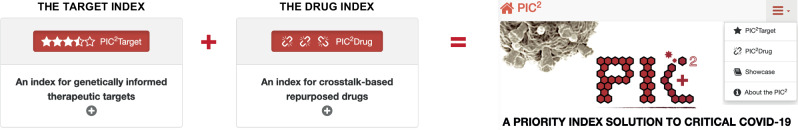
A priority index solution to critical Covid-19. The PIC^2^ resource comprises two indexes: the target index (PIC^2^Target) for genetically informed therapeutic targets, and the drug index (PIC^2^Drug) for crosstalk-based repurposed drugs. Incorporating ‘PI’ (emphasising priority index) and ‘C2’ (symbolising critical Covid-19), the PIC^2^ logo also bears symbols (‘red cross’ and ‘spreading coronavirus’) to signify different interpretations of the letter ‘C’.

Addressing the challenge mentioned above necessitates a paradigm shift in strategies. To this end, we have devised a principled strategy^26,27^ that links non-coding loci to effector genes, eventually cascading down to drug targets. This strategy has found diverse applications for various diseases^28–33^. Building upon our previous accomplishments and aligning with the transition from Covid-19 host genetic findings to translational applications, in this study we report a genetically powered drug-target discovery engine, culminating in the introduction of a resource, namely PIC^2^, made accessible and reproducible via a publicly available web portal (www.genetictargets.com/PIC2) (Fig. 1). The engine features a genetics-led target prioritisation (an approach PIT generating the target index PIC^2^Target), and a crosstalk-based drug repurposing (an approach PID generating the drug index PIC^2^Drug). We demonstrate the validity of the target index in successfully recovering known therapeutics. The drug index highlights opportunities for repurposing clinically approved JAK-STAT inhibitors, either individually or in combination. The introduction of this dual-indexes strategy sets this work apart from our earlier studies^7,34,35^. This strategy is especially distinctive in its capacity to streamline computational translational medicine for Covid-19, seamlessly transitioning from genetic target prioritisation to rational drug repurposing.

## Results

### The target index

**Fig. 2:**
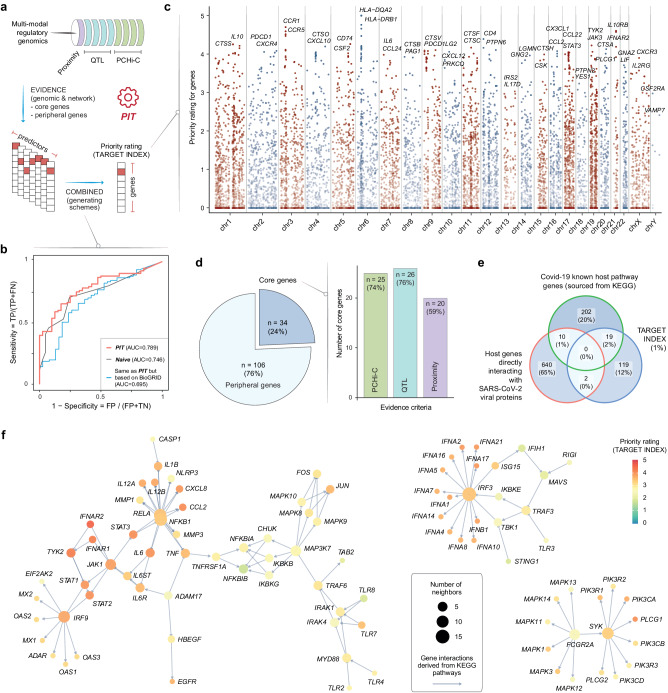
The target index (PIC^2^Target) for critical Covid-19. **a** Target prioritisation workflow. **b** Performance benchmarking. Performance is measured by AUC, distinguishing Covid-19 phase-III drug targets from simulated negative targets. Several target-index generation schemes are compared: PIT (using a logistic meta-analysis combined method and employing network evidence sourced from the STRING database), Naive schemes (frequency of therapeutic targeting by existing licensed/approved drugs, motivated by drug repurposing), and an alternative method employing network evidence sourced from the BioGRID database. Abbreviations include AUC (area under the ROC curve), FN (false negatives), FP (false positives), TN (true negatives), and TP (true positives). **c** Manhattan plot. Priority rating (y-axis) for ~14,000 target genes is illustrated across genomic locations, with the top two prioritised genes per chromosome labelled on the *x*-axis. **d** Pie plot illustrating the composition of core and peripheral genes amongst the top 1% of the target index. Right panel: bar plot for core genes identified through each indicated evidence of criteria. **e** Venn diagram illustrating the overlap between the top 1% of the target index, the Covid-19 host pathway genes (KEGG), and host genes directly interacting with SARS-CoV-2 viral proteins. **f** Network visualisation of the Covid-19 host pathway (sourced from KEGG), with gene nodes color-coded by priority rating and sized by node degree (i.e., the number of neighbours).

As an extension to our previous genetics-led approach^34^, PIT converts host genetic findings (derived from the latest critical Covid-19 GWAS summary-level data^21^) into an index for genetically informed therapeutic targets (Fig. 2a). The target-index generation/prioritisation process involved two key steps (see Materials and Methods): (1) predictor preparation, which harnessed the value of multi-modal regulatory genomic datasets on proximity, quantitative trait locus (QTL), and promoter capture Hi-C (PCHi-C) (constituting genomic evidence), while simultaneously leveraging the knowledge of high-quality protein interactions (sourced from the STRING database^36^) through the random walk with restart (RWR) algorithm (constituting network evidence); and (2) predictor combination, accomplished by benchmarking different target-index generation schemes, including meta-analysis-like schemes (logistic and Fisher’s) and conventional schemes (sum and max). In evaluating how to best combine predictors for generating the target index (Supplementary Fig. 1), it became evident that meta-analysis-like schemes, particularly the logistic scheme, exhibited superior performance over conventional schemes (sum and max), under the same parameter setting for RWR (optimised at the restarting probability of 0.5). We also introduced the Naive scheme as a baseline for evaluation and additionally explored another alternative employing network evidence sourced from the BioGRID database^37^, albeit both alternatives demonstrated inferior performance to PIT (i.e., the logistic scheme anchored by STRING) (Fig. 2b and Supplementary Data 1).

Applying PIT to critical Covid-19 led to the generation of the target index, which involved approximately 14,000 genes, ranked according to their priority rating (Fig. 2c and Supplementary Data 2). To outline this process in more detail, we first used critical Covid-19 GWAS SNPs to define core genes based on evidence drawn from proximity, QTL, and PCHi-C criteria. After establishing core genes, we proceeded to incorporate network evidence obtained from STRING to define peripheral genes. Following this, we computed affinity scores for both core and peripheral genes through RWR, with core genes serving as seed nodes and capitalising on network connectivity. These steps resulted in the preparation of a gene-predictor matrix containing affinity scores for both core and peripheral genes. Next, we used the logistic method to combine the predictors, ensuring the incorporation of both genetic and network evidence. This method, widely employed for combining evidence (*P*-values) from various studies^38^, accommodates the interdependence/non-independence of *P*-values during the aggregation process. By embracing this approach, we combined different predictors grounded in proximity, QTL, and PCHi-C criteria to formulate the target index. The specific steps involved in this process are as follows: (1) for each predictor, the affinity scores resulting from RWR were converted into *P*-like values; (2) these converted *P*-like values were collectively combined across predictors using the logistic method for each gene; and (3) the combined *P*-values were subsequently rescaled to yield a single, unified priority rating (i.e., target index). This unified priority rating ranged from 0 to 5 for each gene (further details provided in Materials and Methods).

In the uppermost (top) 1% of prioritised genes within the target index, approximately three-fourths were genes that did not belong to the core genes (Fig. 2d, left). This highlights a substantial contribution of non-core peripheral genes to the top-ranked genes within our target index, further emphasising the value of incorporating network evidence. To assess the contribution of core genes based on different criteria (proximity, QTL, and PCHi-C), we calculated the proportion contributed by each criterion, with QTL being the most prominent (Fig. 2d, right). Consistent with this, QTL exhibited the most substantial impact on performance when used as the single criterion for prioritisation (Supplementary Fig. 2).

To elucidate the disease relevance of the target index, with a specific focus on the top 1% of prioritised genes, we conducted enrichment analysis using two distinct gene lists (Fig. 2e): (1) a list encompassing human genes/proteins that directly interact with SARS-CoV-2 viral proteins (considering virus-host interactions identified by two or more independent studies^39–45^), and (2) another list comprising Covid-19 human host pathway genes (sourced from KEGG^46^). Amongst the top 1% of prioritised genes, we did not observe any significant enrichment (*P*-value = 0.95 based on Fisher’s exact test) for human proteins that directly interact with SARS-CoV-2 viral proteins (Supplementary Fig. 3). Instead, we found enrichment for Covid-19 host pathway genes. Notably, a substantial proportion of these host pathway genes were present within the top 1% of the target index (odds ratio = 11.1; 95% confidence intervals (CI) = [6.4, 18.4]; *P*-value = 8.0 × 10^−14^). This supports the capacity of the target index in illuminating disease relevance within the context of the human host pathway (Fig. 2f).

**Fig. 3:**
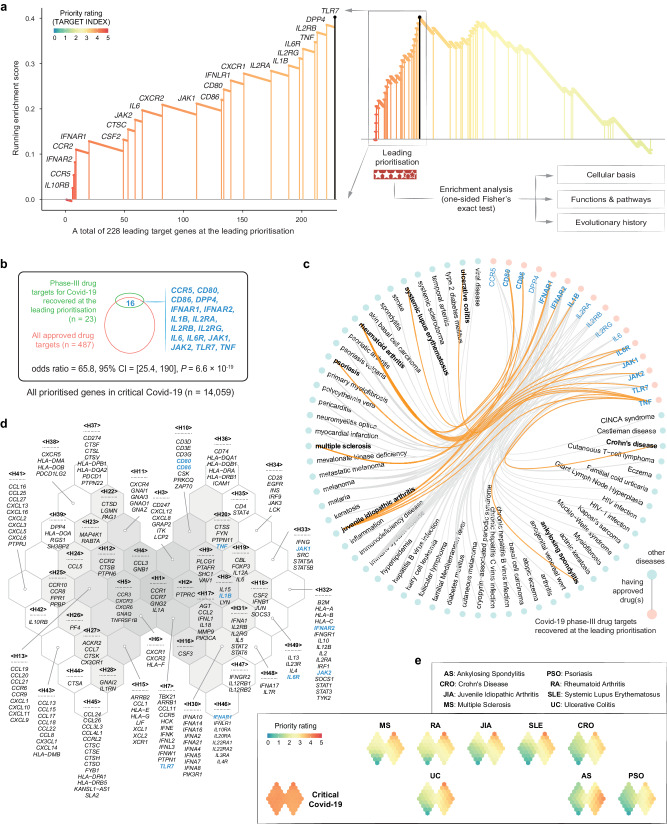
Leading prioritisations for critical Covid-19. **a** Leading prioritisation plot for Covid-19 phase-III drug targets. Those Covid-19 phase-III drug targets (*n* = 23) recovered at the leading prioritisation (zoomed in left panel) are denoted by gene symbols and indicated in vertical lines (also color-coded by priority rating). The leading prioritisation represents the core subset of prioritised target genes that accounts for the enrichment signal, visualised as the left-most region of the peak in the running enrichment plot (middle panel). All genes at the leading prioritisation, referred to as ‘leading target genes’ (*n* = 228), were subjected to enrichment analysis in terms of cellular basis (cell types; see Fig. 4a), functions and pathways (see Fig. 4b), and evolutionary origins (ancestors when they were first created; see Fig. 4c). **b** Venn diagram illustrating the intersection between Covid-19 phase-III drug targets recovered at the leading prioritisation and the entire pool of approved drug targets in any disease indications. The statistical significance (*P*-value), odds ratio, and its 95% confidence intervals (CI; represented by lines) were calculated using one-sided Fisher’s exact test. **c** Hierarchical edge bundling for targets and drugs. Covid-19 phase-III drug targets recovered at the leading prioritisation (in pink) that are already targeted (connected by edges) by approved drugs in other diseases are connected by edges. Those edges involving immune-mediated diseases are highlighted in orange. **d**, **e** Cross-disease prioritisation map between critical Covid-19 and immune-mediated diseases. The map with a 2D butterfly-like topology was trained using a self-organising algorithm for leading target genes in critical Covid-19. **d** The architecture of the map, consisting of 49 hexagons (H1-H49) with genes listed within per hexagon (if any). **e** Disease-specific illustration of target gene prioritisation, with locations in a 2D square lattice capturing inter-disease relationships. This square map was also trained using self-organising algorithm but for diseases.

In addition to its disease relevance, the target index also successfully recovered 23 pre-existing phase-III drug targets for Covid-19 (*P*-value = 5.0 × 10^−5^ based on leading prioritisation analysis; Fig. 3a). These Covid-19 phase-III targets included:

- *IL10RB* (6th) and *IFNLR1* (140th), both targeted by peginterferon lambda-1a (an interferon λ receptor agonist);
- *CCR5* (7th), the target of cenicriviroc and leronlimab (two C-C chemokine receptor Type 5 antagonists);
- *IFNAR2* (8th) and *IFNAR1* (20th), both targeted by interferon beta-1a and peginterferon beta-1a (two interferon α/β receptor agonists);
- *CCR2* (9th), the target of cenicriviroc (a C-C chemokine receptor Type 2 antagonist);
- *CSF2* (49th), targeted by lenzilumab (a GM-CSF inhibitor);
- *CTSC* (53rd), the target of brensocatib (a dipeptidyl peptidase I inhibitor);
- *JAK2* (59th), the target of baricitinib and pacritinib (two JAK2 inhibitors);
- *IL6* (65th), targeted by siltuximab (an interleukin-6 inhibitor);
- *CXCR2* (82nd), the target of reparixin (an interleukin-8 receptor B modulator);
- *JAK1* (111th), the target of baricitinib (a JAK1 inhibitor);
- *CD86* (132nd) and *CD80* (134th), both targeted by abatacept (a T-lymphocyte activation antigen CD86/CD80 inhibitor);
- *CXCR1* (151st), the target of reparixin (an interleukin-8 receptor A modulator);
- *IL2RA* (174th), *IL2RG* (198th), and *IL2RB* (213th), all targeted by aldesleukin (an interleukin-2 receptor agonist);
- *IL1B* (189th), the target of canakinumab (an interleukin-1 β inhibitor);
- *IL6R* (201st), targeted by levilimab, sarilumab, and tocilizumab (three interleukin-6 receptor α subunit inhibitors);
- *TNF* (211th), targeted by adalimumab and infliximab (two TNF-α inhibitors);
- *DPP4* (221st), the target of linagliptin (a dipeptidyl peptidase IV inhibitor); and
- *TLR7* (228th), the target of hydroxychloroquine sulfate (a Toll-like receptor 7 antagonist).

Notably, baricitinib, a selective oral JAK1/2 inhibitor, has been shown to improve the use of remdesivir, the only FDA-approved antiviral drug for the treatment of Covid-19 (ref. ^47^). Baricitinib not only reduces recovery time but also accelerates improvement in clinical status^48^. The REMAP-CAP randomised clinical trial revealed that critical Covid-19 patients receiving IL6 receptor inhibitors (tocilizumab or sarilumab) exhibited an improved 180-day mortality rate^49^. Collectively, genes identified at the leading prioritisation (referred to as ‘leading target genes’ hereinafter; listed in Supplementary Data 3) tended to be therapeutically considered for targeting to treat Covid-19. Approximately 70% (16/23) of these genes have already been targeted by approved drugs in other diseases (odds ratio = 65.8; 95% CI = 25.4, 190); *P*-value = 6.6 × 10^−19^; see Fig. 3b), especially in immune-mediated diseases. These immune diseases include ankylosing spondylitis, Crohn’s disease, juvenile idiopathic arthritis, multiple sclerosis, psoriasis, rheumatoid arthritis, systemic lupus erythematosus, and ulcerative colitis (Fig. 3c). Moreover, when incorporating our previous findings^34^, we found that these approved drug targets were also highly prioritised in their respective immune diseases, including *CD80/86*, *IFNAR1/2*, *IL1B*, *IL6R*, *JAK1/2*, *TLR7*, and *TNF* (Fig. 3d, e and Supplementary Data 4).

**Fig. 4:**
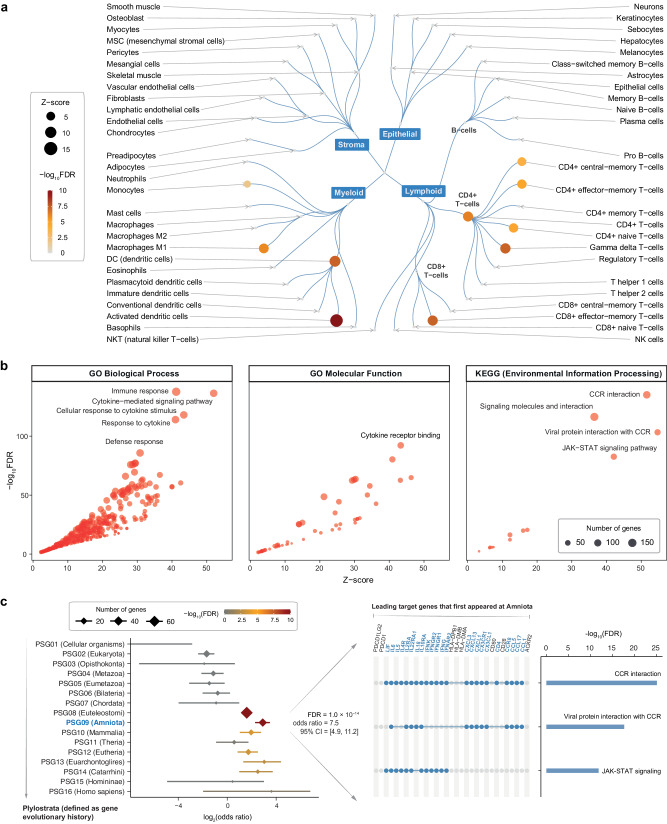
Enrichment analysis for leading target genes. **a** Circular illustration of cell type enrichments, with nodes sized by *Z*-score and color-coded by FDR. **b** Scatter plot illustrating enriched functions and pathways, with the 10 most significant terms/pathways labelled. Functions are based on Gene Ontology (GO) Biological Process and Molecular Function terms, while pathways based on KEGG (Environmental Information Processing pathways). Each dot corresponds to an individual term/pathway, with the size proportional to the number of its member genes. **c** Forest plot illustrating enriched phylostrata (ancestors) ordered by evolutionary history. Genes first created at the ancestor ‘Amniota’ are indicated in the right panel, along with their pathway enrichments. Notably, the significance level (FDR), odds ratio, and its 95% confidence intervals (CIs; represented by lines) were calculated using one-sided Fisher’s exact test.

**Fig. 5:**
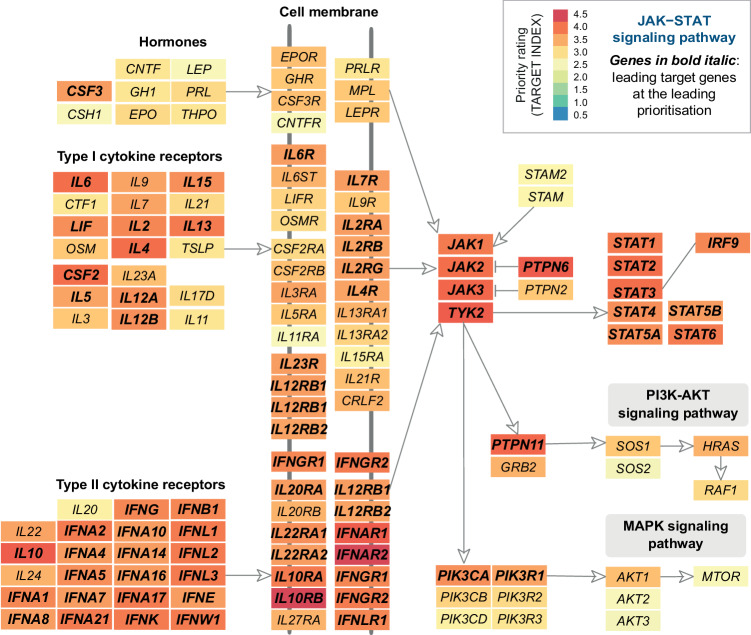
JAK-STAT signaling pathway. Its member genes are color-coded by priority rating, with leading target genes highlighted in bold.

Based on leading target genes, we delved deeper into: (1) the cellular basis, revealing the involvement of multiple immune and inflammatory lineages (Fig. 4a and Supplementary Fig. 4); (2) molecular functions, biological processes, and environmental information processing pathways (Fig. 4b and Supplementary Data 5); and (3) their evolutionary origins, particularly emerging at the time of Amniota (the most recent common ancestor of all living reptiles, birds, and mammals) (Fig. 4c and Supplementary Data 6). Amniotes represent the clade of tetrapod (‘four-leg’) vertebrates characterised by protective extra-embryonic membranes. Remarkably, genes originating at Amniota were mostly involved in cytokine-cytokine receptor interactions, as well as the JAK-STAT signaling (i.e., *IFNAR2*, *IFNG*, *IFNGR1/2*, *IFNK*, *IL10RA*, *IL22RA1*, *IL2RA*, *IL4R*, *IL5/6*, and *LIF*). The knowledge of molecular interactions within the JAK-STAT signaling was illustrated, with genes color-coded based on their priority rating (Fig. 5).

### The drug index

**Fig. 6:**
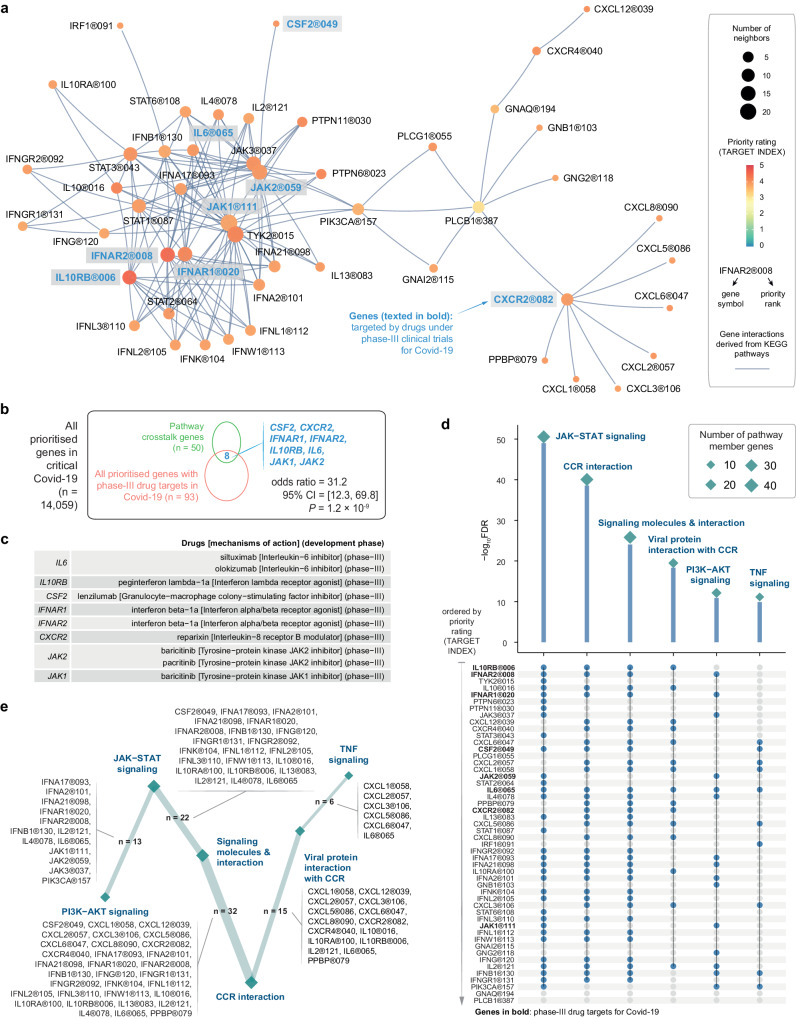
Pathway crosstalk for critical Covid-19. It was identified from pathway-derived gene interactions but constrained by target gene prioritisation information (i.e., target index). **a** Gene-centric representation of the crosstalk, with nodes labelled by ‘gene symbol @ priority rank’ and color-coded by priority rating (target index). The crosstalk involves highly prioritised and interconnected genes. Genes targeted by drugs in phase-III clinical trials for Covid-19 are highlighted in bold. **b** Venn diagram illustrating the overlap between crosstalk genes and Covid-19 phase-III drug targets. The statistical significance (*P*-value), odds ratio, and its 95% CI were calculated using one-sided Fisher’s exact test. **c** Tabular display of crosstalk genes also being Covid-19 phase-III drug targets, along with information on drug candidates and mechanisms of action. **d** Kite-like plot for KEGG pathways enriched in crosstalk genes. Enrichment significance (FDR) was calculated using one-sided Fisher’s exact test. Each kite is sized by the number of member genes (indicated by blue dots beneath). Abbreviations include CCR (cytokine-cytokine receptor) and TNF (tumor necrosis factor). **e** Pathway-centric representation of the crosstalk, with node size indicating the number of pathway member genes, and edge thickness proportional to the number of shared genes between two-endpoint pathways.

One of the distinguishing features of PID lies in its capacity to perform crosstalk-based drug repurposing and effect-by-removal analysis to determine the impact of node removal on pathway crosstalk, generating an index for crosstalk-based repurposed drugs (refer to Fig. 1c). Central to this capability is the identification of genes that mediate crosstalk between pathways. The resulting 50-gene network for pathway crosstalk (*P*-value = 1.41 × 10^−112^ based on the permutation test) all contained highly prioritised genes in critical Covid-19 (Fig. 6a and Supplementary Data 7). Remarkably, 8 genes (i.e., *CSF2*, *CXCR2*, *IFNAR1/2*, *IL10RB*, *IL6*, and *JAK1/2*) targeted by drugs currently undergoing phase-III clinical trials for Covid-19 were present within this network (Fig. 6b, c). By focusing on pathways significantly over-represented in this crosstalk network (Fig. 6d and Supplementary Data 8), we could represent the crosstalk at the pathway level, with edges estimated based on the extent of genes being shared between two endpoint pathways (Fig. 6e).

We proceeded to explore the evidence in support of drug repurposing by asking whether pathway crosstalk genes could be targeted by approved or phased drugs in diseases other than Covid-19. Our analysis yielded substantial clinical evidence (approved drugs) with false discovery rate (FDR) of 1.9 × 10^−8^, identifying 13 genes already targeted by approved drugs used to treat other diseases, that is, licensed medications in clinical use for diseases other than Covid-19 (Supplementary Fig. 5). Notably, out of these 13 approved drug targets, 12 genes (*IFNAR1/2*, *IFNG*, *IFNGR1/2*, *IL6/13*, *JAK1/2/3*, *PIK3CA*, and *TYK2*) were integral to the JAK-STAT signaling pathway, highlighting repurposing opportunities of targeting this pathway. Additionally, we identified four phase-III drug targets (*CSF2*, *CXCR2*, *IL10RA*, and *IL10RB*; FDR = 5.9 × 10^−3^).

**Fig. 7:**
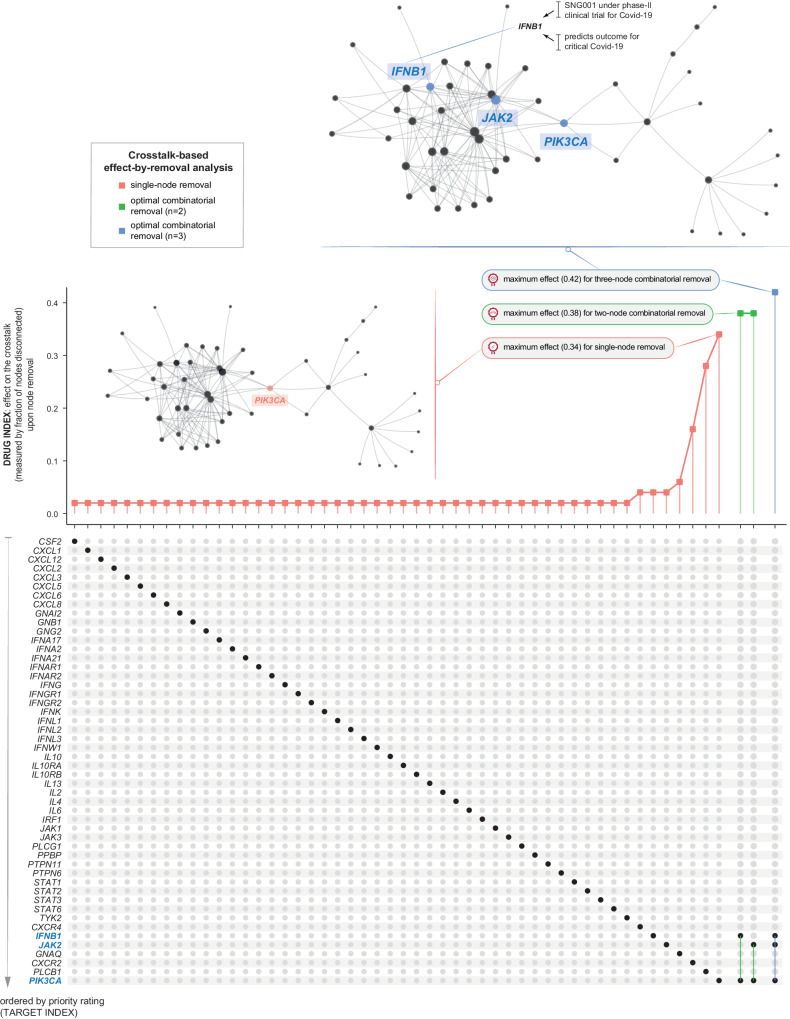
The drug index (PIC^2^Drug) for critical Covid-19. Effect of node removal, either individually or in combination, on the crosstalk. The fraction of disconnected nodes (i.e., the drug index) on the *y*-axis is plotted against node removal (indicated by solid circles beneath) on the *x*-axis. Notably, only the optimal removal with the largest effect is illustrated for two- or three-node combinatorial removal. Inserted is visualisation of the crosstalk, with the same layout as Fig. 6a, but labelled only for genes with optimal removals.

Finally, we delved into drug repurposing opportunities by implementing crosstalk-based effect-by-removal analysis. Effect-by-removal analysis gauged the vulnerability of the pathway crosstalk to node removal, either individually or in combination; removing critical network nodes would result in a larger proportion of disconnected nodes in the crosstalk (Fig. 7 and Supplementary Data 9). This analysis generated an index for repurposed drugs, that is, the drug index measured as the fraction of disconnected nodes (ranged 0–1). The drug index underscored repurposing opportunities of clinically approved inhibitors targeting the JAK-STAT signaling. Notably, removing the gene *PIK3CA* alone resulted in a maximum of 36.4% node disconnection (i.e., the drug index of 0.34). This gene is intricately linked to the PI3K/AKT/mTOR axis and presents a potential Covid-19 pharmacological target^50^. The disconnection fraction increased to 40.0% when further removing *JAK2* (i.e., *PIK3CA* + *JAK2*) or *IFNB1* (i.e., *PIK3CA* + *IFNB1*), representing the maximal/optimal effect achievable by removing any two nodes in combination (i.e., the drug index of 0.38). Further disconnection, reaching a maximum of 42% disconnections (i.e., the drug index of 0.42), was observed upon removing *PIK3CA* + *JAK2* + *IFNB1*. In summary, drug repurposing coupled with effect-by-removal analysis provided evidence for targeting key components (i.e., *PIK3CA*, *JAK2*, and *IFNB1*) of the JAK-STAT signaling pathway, either individually or in combination (potentially for the poly-therapeutics discovery in critical Covid-19)^51^. The ongoing focus on targeting this pathway is reinforced by the presence of therapeutic agents now in active phase-II/III clinical trials in Covid-19. This includes therapeutic agents targeting the gene *IFNB1*, which are currently under phase-II clinical trials for Covid-19 (Fig. 7, top), while agents targeting the gene *JAK2* are undergoing phase-III trials for Covid-19 (also see Fig. 6c).

## Discussion

In deepening our long-standing efforts in therapeutic target prediction and validation, our work has culminated in the creation of the PIC^2^ solution, dedicated to expediting Covid-19 translational medicine research. It streamlines the running from genetic target prioritisation to rational drug repurposing, thereby contributing to informed decisions about potential therapeutic candidates for next preclinical validations and, ultimately, clinical trials.

An outstanding feature of PIC^2^ lies in its capacity in seamlessly integrating GWAS summary-level data with a wealth of multi-modal regulatory genomic datasets and high-quality protein interactions. Comprehensively incorporating both genetic and network information, this integration culminates in the generation of the target index, demonstrated to be informative in recovering pre-existing Covid-19 phase-III drug targets. Another notable feature offered by PIC^2^ is its capacity in discerning crosstalk between pathways, which represents an advancement compared to our previous studies^34,35^. This crosstalk-based approach enhances the identification of genes for potential drug repurposing. The effect-by-removal analysis is an addition that provides insights into the impact of removing specific nodes (genes) from the network, allowing for the evaluation of the consequences of targeting individual genes or their combinations, particularly in the context of pathway crosstalk, which is essential for drug repurposing. This crosstalk-based drug repurposing, when coupled with effect-by-removal analysis, is a component that enhances the methodology’s depth and applicability. As exemplified in this study, it offers an avenue for repurposing clinically approved JAK-STAT inhibitors, whether as single agents or in combination, for potential application in the treatment of severe Covid-19 cases.

Acknowledging the limitations of PIC^2^ is crucial, particularly in its reliance on the availability and quality of regulatory genomic datasets. The quality and abundance of these datasets play a pivotal role in establishing links between non-coding loci and the core genes that underlie host genetic associations. As QTL and PCHi-C datasets continue to expand across diverse cell types, states, and tissues, improvements in the accuracy and comprehensiveness of target prioritisation and drug repurposing are anticipated. Exploring alternative rating schemes, such as Brown’s method^52^ and the Cauchy combination test^53^, offers a potential avenue to explicitly address the challenge of interdependence/non-independence between predictors. Another limitation arises from the lack of extensive experimental or clinical validations, even though we have demonstrated the validity and utility of the leading prioritisation and drug repurposing. The dual indexes for targets and drugs form the foundation for validation and repurposing within the wider research community. However, the need for substantiated therapeutic efficacy remains paramount and necessitates further evidence, potentially derived from human disease-relevant assays.

Our study, primarily oriented toward the prioritisation of potential therapeutic targets for critical Covid-19, does not explicitly determine the specific directionality (i.e., agonism or antagonism) of the prioritised genes. Instead, the prioritisation stems from the perceived relevance of these genes within the disease context. Additional experimental and functional investigations are required to ascertain the precise directionality of therapeutic intervention (agonism or antagonism) for prioritised genes. Such studies encompass in vitro and in vivo assays, preclinical models (particularly human organoids^54,55^), and clinical trials, enabling a comprehensive understanding of the effects resulting from the modulation of the activity of these genes. Our approach yields a prioritised list of potential therapeutic targets, mandating further empirical validation to define the most optimal therapeutic intervention strategy.

The development of the PIC^2^ solution represents a big step forward in the realm of critical Covid-19 research. However, it is just the beginning of an exciting journey toward more accurate, comprehensive, and context-specific target prioritisation and validation. We are committed to embracing the potential future directions (detailed below) and welcome collaborative efforts to realise these objectives. By integrating omics data, utilising functional annotations, and considering context-specific interactions, we aim to enhance our understanding of Covid-19 mechanisms and improve the precision of target prioritisation, ultimately contributing to the development of effective therapeutic strategies for critical Covid-19 patients.

- Integration of omics data. One of the most promising areas for future exploration involves the integration of diverse omics data, including transcriptomic, epigenomic, and proteomic information, which can enhance the comprehensiveness of the target index. While our study primarily focused on genetic and network evidence (which is less context-specific), we recognise the significance of including these additional layers of omics data information to further refine and validate the target index. The inclusion of cell-type-specific expression information, for instance, could facilitate the selection of prioritised targets in specific cell types for experimental validation. This context-specific data is vital for refining the prioritisation of genes for therapeutic targeting. Additionally, the integration of epigenomic and proteomic data can provide insights into post-transcriptional and post-translational modifications, offering a more comprehensive understanding of target functionality. By combining these data types, future research can achieve a deeper comprehension of critical Covid-19 mechanisms and enhance therapeutic target prioritisation.

- Utilisation of functional annotations. The incorporation of functional annotations, such as gene ontology terms and curated pathway information, is another avenue for improving target prioritisation. By associating target genes with specific biological processes or pathways relevant to Covid-19, this approach can enhance the contextualisation of the relationship between gene function and disease progression. Curated pathways from reputable databases, like KEGG, can serve as reliable sources to guide the functional annotation process. This additional layer of data can refine the selection of prioritised targets in specific biological contexts, thus increasing the precision of target prioritisation.

- Context-specific interactions. The importance of considering context-specific interactions, especially tissue-specific or cell-type-specific interactions, cannot be overstated. Accurately representing host-virus interactions necessitates leveraging context-specific networks to obtain a more precise understanding of the disease process within the context of critical Covid-19. These context-specific interactions are invaluable for identifying critical nodes within specific cell types or tissues that play pivotal roles in the disease process. As the importance of context-specific information grows in phenomics^56,57^, our future endeavors will delve deeper into this aspect.

In summary, our PIC^2^ solution, tailored for critical Covid-19, offers a platform for in silico and timed translation of host genetic findings into drug repurposing. The accessibility, reproducibility, and the potential for scalability make it a robust solution. Looking beyond the scope of this study, the dual-indexes strategy holds promise for scalability, positioning it as an adaptable approach for addressing a wide range of disease domains. Within these domains, computational translational medicine emerges as an indispensable tool, swiftly bridging the gap between host genetic insights and their effective applications in translational therapeutics.

## Materials and methods

### Generating an index for genetically informed therapeutic targets for critical Covid-19 (PIC^2^Target) through a genetics-driven and network-based approach (PIT)

The creation of the index involved the implementation of our previously established multi-step integrative prioritisation strategy, which integrates a genetics-driven and network-based approach^26,34,35^. To elucidate, we used critical Covid-19 GWAS summary-level data^21^ as inputs and harnessed both genetic evidence (from multi-modal regulatory genomic datasets) and network evidence (from high-quality protein interactions), to output a comprehensive list of approximately 14,000 target genes, ranked by their priority ratings on a scale of 0–5. This intricate prioritisation procedure comprises the following key components.

- GWAS SNPs. We input GWAS-detected SNPs with a significant threshold of *P*-value < 5 × 10^−8^. In addition, SNPs in linkage disequilibrium (LD; R^2^ >= 0.8) were considered according to the European population^58^. The scoring formula for SNPs accounted for various aspects of disease genetic associations, including p-values, the significance threshold, and R^2^ values.

- Core genes. SNPs scored above were used to define core genes through evidence of genomic proximity (nearby genes), e/pQTL (QTL genes)^59–65^, and PCHi-C (conformation genes)^66–71^. In addition to SNP scores, the scoring for core genes also encompassed the following factors: (1) the distance-to-SNP window for genomic proximity; (2) the significance level of genetic associations with gene expression for eQTL datasets or protein abundance for pQTL datasets; and (3) the strength of gene promoters physically interacting with SNP-harbouring genomic regions for PCHi-C datasets. The empirical cumulative distribution function (eCDF) was estimated based on all SNP-gene pairs to ensure scaling within the 0–1 range.

- Peripheral genes. Using core genes as seeds, we implemented the RWR algorithm to identify (non-seed) peripheral genes under network influence through exploiting knowledge of protein interactions. The RWR algorithm is a propagation-based method that simulates the information flow within a network to estimate connectivity (affinity) between two nodes. This method assigned affinity scores to genes based on their connectivity to a set of ‘seed’ genes (in this context, core genes identified through genetic evidence). We established a network of protein interactions primarily from the STRING database^36^, incorporating interactions with an interaction score no less than 0.7 (i.e., high confidence) and labelled as ‘experiments’ and ‘databases’ (i.e., manually curated). This interaction network encompassed approximately 14,000 nodes/genes (and around 201,000 interactions/edges). There were two types of nodes in this gene interaction network: nodes labelled as seed genes (along with their corresponding scores as weights), and the remaining nodes as non-seed genes. The algorithm started a random walk from each seed gene and iteratively traversed neighboring genes along network edges. At each iteration, the walker faced a choice: either moving to a randomly chosen neighbor or jumping back to the seed node. This process continued until a steady state was reached, characterised by the probability distribution of each gene stabilising. The resulting steady probability values, ranging from 0 to 1, stored affinity scores for each gene relative to seed core genes. For each of datasets (i.e., proximity, QTL, and PCHi-C), the process above yielded a predictor containing core and peripheral genes, accompanied by affinity scores that quantified their network connectivity to seed core genes. Notably, genes with higher network connectivity to seed core genes were assigned higher affinity scores. Generally, seed genes were more likely to receive higher affinity scores than non-seed peripheral genes. Nonetheless, a peripheral gene might receive a high affinity score if it exhibited high connectivity to most (if not all) seed genes. Thus, non-seed peripheral genes with higher affinity scores denoted genes highly influenced by the network.

- Gene-predictor matrix. Employing the abovementioned multi-step scoring procedures (GWAS/LD SNPs – core genes – peripheral genes), a gene-predictor matrix was constructed. This matrix featured rows for genes and columns for predictors. Within this matrix, affinity scores were amalgamated, collectively incorporating genetic evidence and network evidence.

- Performance benchmarking for target-index generation/prioritisation schemes. Leveraging the gene-predictor matrix constructed above, various generation schemes were evaluated for combining predictors. Performance comparisons were conducted between (1) meta-analysis-like schemes, including logistic and Fisher’s combined methods (detailed in the subsequent section ‘Gene-level prioritisation’), and (2) conventional schemes, comprising sum (additively aggregating affinity scores across predictors) and max (selecting the maximum one across predictors). Performance was also compared with a Naive scheme, wherein genes were prioritised based on the frequency of being therapeutically targeted by licensed/approved drugs. This Naive prioritisation, motivated by drug repurposing (though not applicable for prioritising new targets), served as the *status quo* baseline for target-index prioritisation. Performance assessment relied on the area under the ROC curve (AUC) for distinguishing Covid-19 phase-III drug targets from simulated negative target controls. Information on phase-III drug targets in Covid-19 was sourced from the ChEMBL database^72^, encompassing drug candidates and mechanisms of action explaining drug efficacy. Simulated negative target controls for Covid-19 were established based on known therapeutics from ChEMBL, involving three steps: (1) defining the druggable landscape as all known target genes across various diseases and drug development phases; (2) extracting Covid-19 drug targets (regardless of drug development phase) from ChEMBL, along with their interacting neighbours determined by interaction information from databases (STRING^36^ and Pathway Commons^73^); and (3) simulating negative targets as genes from the druggable landscape after excluding Covid-19 drug targets and their interacting neighbours.

- Target-index generation/prioritisation. For comprehensive mathematical explanations regarding Fisher’s combined meta-analysis method, please consult our previous publication^34^. Building upon the performance evaluation elucidated in the previous section, in this study, PIT used a logistic combined meta-analysis method to consolidate predictors within the gene-predictor matrix prepared earlier. Within the gene-predictor matrix, affinity scores for a given predictor were first converted into *P*-like values using empirical cumulative density function (eCDF) estimated from affinity scores of all genes within that predictor (Eq. 1).

1 $${P}_{i}^{j}={eCDF}({{AF}}_{i}^{j}),$$

Here, ${{AF}}_{i}^{j}$ denotes the affinity score for the *ith* gene concerning the *jth* predictor, ${P}_{i}^{j}$ is the corresponding converted *P*-value, and *eCDF* is estimated based on all genes.

Subsequently, converted *P*-values for a gene were combined across predictors via a logistic combined method (Eqs. 2–4*)*; see the publication^38^ for a more detailed explanation. Ultimately, the combined *P*-value underwent rescaling to yield priority rating (continuously from 0–5 and corresponding to priority rank from 1 to ~14,000). This output is referred to as an index for target genes (denoted as PIT; Eq. 5).

2 $${x}_{i}=-2{\sum }_{j}^{J}\left[{P}_{i}^{j}/\left({1-P}_{i}^{j}\right)\right],$$

3 $${x}_{i} \sim {St}\left(5J+4\right),$$

4 $${{CP}}_{i}={CDF}({x}_{i}),$$

5 $${{PIT}}_{i}=5\times \frac{-\log {{CP}}_{i}-{{MIN}}_{k}^{K}\left(-\log {{CP}}_{k}\right)}{{{MAX}}_{k}^{K}\left(-\log {{CP}}_{k}\right)-{{MIN}}_{k}^{K}\left(-\log {{CP}}_{k}\right)},$$

In these equations, ${P}_{i}^{j}$ denotes the converted *P*-value for the *ith* gene within the *jth* predictor, *J* is the number of predictors, *St*(5*J+*4) denotes Student’s t-distribution with *5J* *+* *4* degrees of freedom, *CP*_*i*_ symbolises the combined *P*-value for the *ith* gene (that is, CDF valued at *x*_*i*_), and *PIT*_*i*_ represents the priority rating for the *ith* gene (among *K* genes, where *K* is ~14,000).

- Analysis using human genes/proteins that directly interact with SARS-CoV-2 viral proteins. Interactions between SARS-CoV-2 viral proteins and human proteins were obtained from the BioGRID database (version 4.4.214)^37^. A total of 2637 virus-host interactions, encompassing 29 SARS-CoV-2 viral proteins and 652 human proteins, were independently identified by two or more studies^39–45^. Fisher’s exact test was employed to estimate the significance of the overlap between virus-interacting human proteins and the top 1% of prioritised genes. This test was also performed for human proteins interacting with each of the 29 SARS-CoV-2 viral proteins.

- Analysis using known Covid-19 human host pathway. The Covid-19 human host gene interaction pathway, expertly curated and extensively documented in www.genome.jp/pathway/hsa05171 as part of the KEGG database^46^, focuses notably on the downstream effects of the SARS-Cov-2 virus on the host. These effects involve not merely direct interactions with host proteins, but also encompass various downstream outcomes such as the activation of the NF-kB pathway, IL-6 production, systemic inflammation, and many others. Retrieving this information from KEGG involved utilising the KEGGgraph package (version 1.56.0) to download the KGML-formatted file, which was subsequently processed and converted into an igraph R object. Fisher’s exact test was used to assess the significance of the overlap between the top 1% prioritised genes and the genes associated with the host pathway. The stress majorisation algorithm was applied to visualise the network layout of gene interactions within the host pathway.

- Leading prioritisation. The concept of leading prioritisation pertains to the core subset of all prioritised genes, conveniently referred to as ‘leading target genes’, which contribute to the enrichment of phase-III drug targets in Covid-19. This enrichment was visually presented as the leftmost region of the peak within the running enrichment plot generated by target set enrichment analysis. The dnet package (version 1.1.7)^74^ facilitated this analysis by quantifying the degree to which Covid-19 phase-III drug targets were enriched at the leading prioritisation. Essentially, Covid-19 phase-III drug targets found at the leading prioritisation were assigned high priority. The significance (*P*-value) of the enrichment was ascertained through permutation tests conducted 50,000 times.

- Cross-disease prioritisation map. We employed a self-organising algorithm implemented in the supraHex package (version 1.40.0)^75–77^ to construct a cross-disease prioritisation map for leading target genes in critical Covid-19. A butterfly-shaped map, consisting of *N* = 49 hexagons, was trained using the input prioritisation matrix (containing priority rating) across *M* = 9 diseases. These diseases included critical Covid-19 (this study) and 8 immune-mediated diseases^34^: ankylosing spondylitis, Crohn’s disease, juvenile idiopathic arthritis, multiple sclerosis, psoriasis, rheumatoid arthritis, systemic lupus erythematosus, and ulcerative colitis. The codebook matrix associated with the trained map was utilised to provide a disease-specific view of target gene prioritisation. To further visualise inter-disease relationships, a 2D square map lattice was used to self-organise diseases in a manner that geometric locations within this square lattice delineated the relationships between diseases.

- Enrichment analysis for leading target genes. OpenXGR^78,79^ was used for conducting enrichment analysis on leading target genes to identify over-representation (i.e., enrichments) in terms of cellular basis (cell types), functions (GO terms), pathways (KEGG pathways), and evolutionary history (phylostrata). Cell-type-specific gene signatures were extracted from xCell^80^. GO Biological Process and Molecular Function terms, alongside their annotated genes, were obtained from NCBI^81^. Environmental Information Processing (EIP) pathways and their member genes were sourced from KEGG^46^, which covers a diverse range of molecular pathways, including EIP and six other categories. Each category captures its unique knowledge domain of molecular interactions with relative completeness. Genes first created at each of 16 phylostrata were extracted from this study^82^ to delve into the evolutionary history of leading genes. Enrichment analysis was performed using one-sided Fisher’s exact test, producing FDR, *Z*-scores, odds ratio and its 95% CI. A bipartite graph was crafted to illustrate the relationship between enriched cell types and their member genes.

### Generating an index for repurposed drugs in critical Covid-19 (PIC^2^Drug) through pathway crosstalk-based effect-by-removal analysis (PID)

- Identification of pathway crosstalk. This identification focused on genes with high ratings and strong interconnectedness. The search involved selecting a subset of gene interactions that were merged from KEGG EIP pathways. The intention of choosing EIP pathways aimed to emphasise molecular interactions involved in signal transductions that are less organism, cell type, and disease- specific. These EIP pathways encompassed 2158 genes, collectively forming a network of 15,375 interactions between these genes. Each interaction in this gene network was present in at least one EIP pathway(s). The process of identifying pathway crosstalk utilised a heuristic solution for solving the prize-collecting Steiner tree problem^74,78^. To assess the statistical significance (*P*-value) of the identified crosstalk, a degree-preserving node permutation test was conducted with 100 iterations. Additionally, the analysis allowed for the specification of a desired number (e.g., 50) of nodes or genes in the resulting crosstalk could be specified, and this desired output was obtained through a well-established iterative search procedure. For comprehensive details, please refer to our previous publications^74,78,79^. The identified crosstalk, apart from its portrayal as a gene network, was also visualised as a pathway-centric network, in which pathways were depicted as nodes and their inferred connections as network edges. Only pathways that exhibited significant over-representation in crosstalk genes, as determined by a one-sided Fisher’s exact test, were included as nodes. The edges were initially inferred based on the shared member genes between pathways, and then filtered by identifying the minimum spanning tree using the igraph package (version 1.6.0). This process retained only the edges present within the resulting tree, with the thickness of edges adjusted proportionally according to the number of shared member genes between the two endpoint pathways.

- Crosstalk-based drug repurposing. The drug repurposing analysis relied on information extracted from ChEMBL^72^, which aggregated therapeutic data on current phase-III and approved therapeutics (including drugs, development phases, target genes and disease indications). For a disease indication, drugs reaching the maximum phase of development were selected for a target gene, considering that selected target genes had well-defined mechanisms of action and could explain the efficacy of drugs in treating the disease. These selected target genes were categorised into two distinct groups: one encompassing approved drug targets (i.e., genes targeted by any approved drugs), and the other comprising phased drug targets (i.e., genes targeted by non-approved phased drugs that are in developmental phases, not by any approved drugs). One-sided Fisher’s exact test was used to evaluate the statistical significance of crosstalk genes enriched for two drug target groups (i.e., approved drug targets and phased drug targets).

- Crosstalk-based effect-by-removal analysis. The removal analysis was designed to evaluate the effect of nodes on the crosstalk. This analysis comprised two dimensions: (i) the individual removal of nodes (i.e., single-node removal), and (ii) the simultaneous removal of nodes in combination (i.e., combinatorial removal). Single-node removal was undertaken to define an index for repurposed drugs targeting the node being removed. In instances where a specific node earmarked for removal was pivotal for the network, its removal would result in the disconnection of a substantial fraction of nodes from the largest network component. This fraction, embodying the number of disconnected nodes, was used to quantify the drug index. Combinatorial removal, on the other hand, sought to select optimal combinations for targeting, with the objective of maximising the effect resulting from the simultaneous removal of specific node combinations (e.g., removing two nodes at once). In other words, the largest fraction of disconnected nodes was sought for removing a specific node combination. The effect of node removal was visually captured through an upset plot using the ggupset package (version 0.3.0). This visualisation method allowed us to clearly depict the outcomes of both single-node and combinatorial node removal.

### Statistics and reproducibility

All statistical analyses were performed using R (version 4.3.0), with one-sided Fisher’s exact test used for performing enrichment analysis, reporting the statistical significance, odds ratio, and its 95% confidence intervals. In the spirit of supporting reproducibility, we offer showcases (http://www.genetictargets.com/PIC2/showcase) that enable users to reproduce all findings presented in this study. These showcases include input data, line-by-line codes, as well as tabular and graphical outputs, all embedded into a single self-contained HTML file. Within this file, users can find comprehensive, step-by-step instructions guiding them through showcases and providing a preview of anticipated results.

### Reporting summary

Further information on research design is available in the Nature Portfolio Reporting Summary linked to this article.

### Supplementary information

Supplementary Figs. 1-5

Supplementary Data 1

Supplementary Data 2

Supplementary Data 3

Supplementary Data 4

Supplementary Data 5

Supplementary Data 6

Supplementary Data 7

Supplementary Data 8

Supplementary Data 9

Reporting Summary

## Data Availability

Source data for graphs within this paper are provided in the Supplementary Data files. More specifically, source data in Supplementary Data 1 are used to generated Fig. 2b, source data in Supplementary Data 2 for generating Fig. 2c, source data in Supplementary Data 3 for generating Fig. 3a, source data in Supplementary Data 4 for generating Fig. 3d, e, source data in Supplementary Data 5 for generating Fig. 4b, source data in Supplementary Data 6 for generating Fig. 4c, source data in Supplementary Data 7 for generating Fig. 6a, source data in Supplementary Data 8 for generating Fig. 6d, and source data in Supplementary Data 9 for generating Fig. 7. Together with these source data, data used and generated during the current study are also accessible through a web-based open-access portal at http://www.genetictargets.com/PIC2. This portal empowers users to interactively explore two indexes comprising genetic targets and repurposed drugs for critical Covid-19.

## Code availability

Software codes have been encapsulated within an R package, accessible at https://hfang-bristol.github.io/PIC2. Additionally, codes have been deposited into a public data repository at Figshare (10.6084/m9.figshare.24967797).
