# Supplementary material for: Priority index for critical Covid-19 identifies clinically actionable targets and drugs
Source: Commun Biol. 2024 Feb 16;7:189. doi: 10.1038/s42003-024-05897-0 (PMC10873402; doi:10.1038/s42003-024-05897-0)
Supplement: Supplementary file 1 — Supplementary Figs. 1-5 [file 42003_2024_5897_MOESM1_ESM.pdf]

# πSupplementary Figures

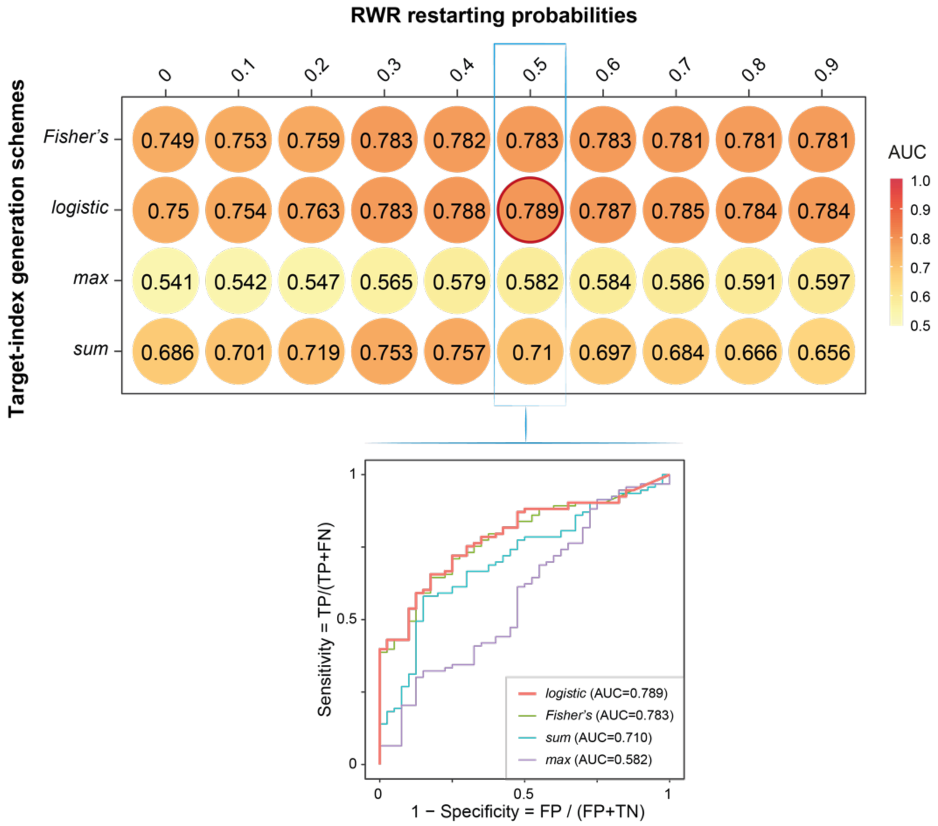


**Supplementary Figure 1: Comparing the target-index generation schemes, including the optimisation of the RWR restarting probability.** AUC plots demonstrate performance comparison among different schemes at the optimised restarting probability (i.e., 0.5).

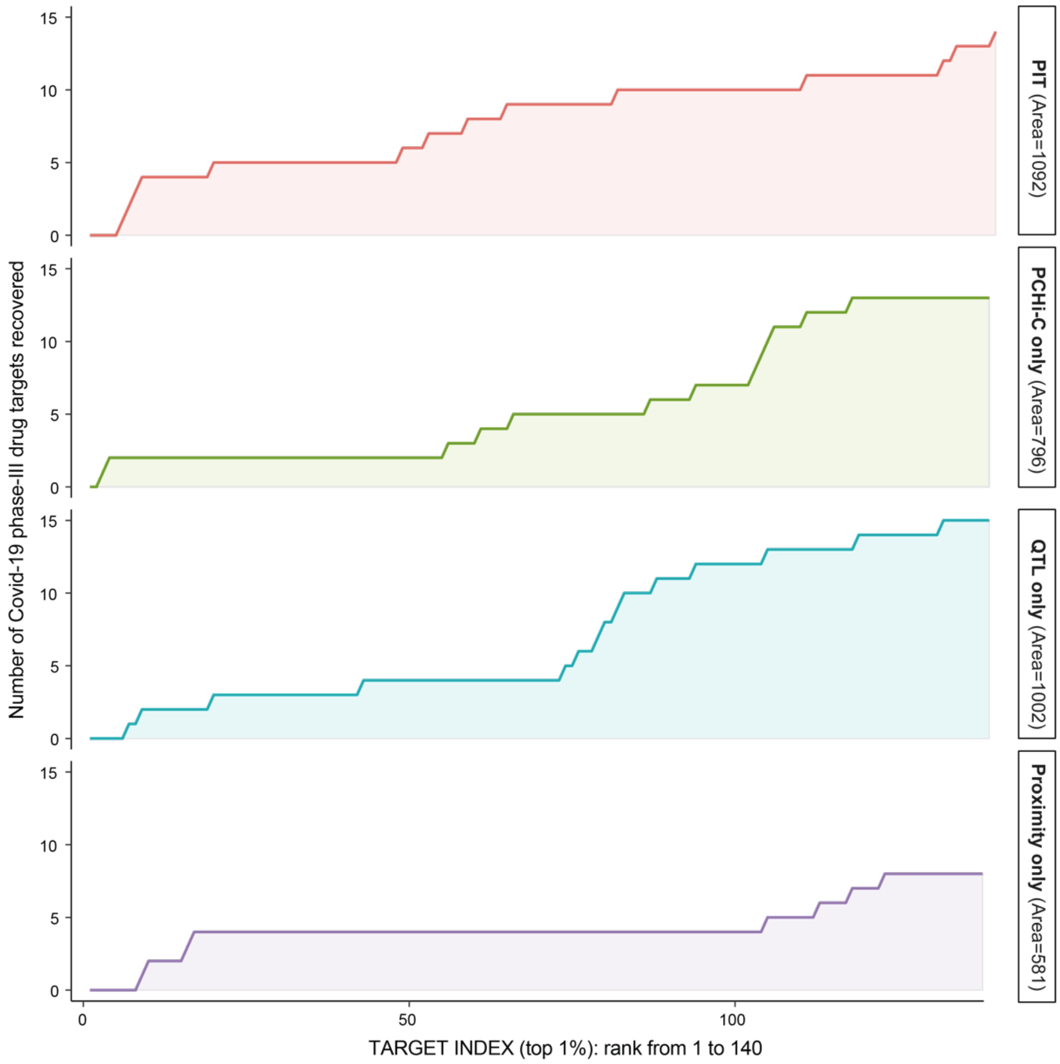


**Supplementary Figure 2: Evaluation of Covid-19 pre-existing phase-III drug target recovery by prioritisation using single criterion evidence.**

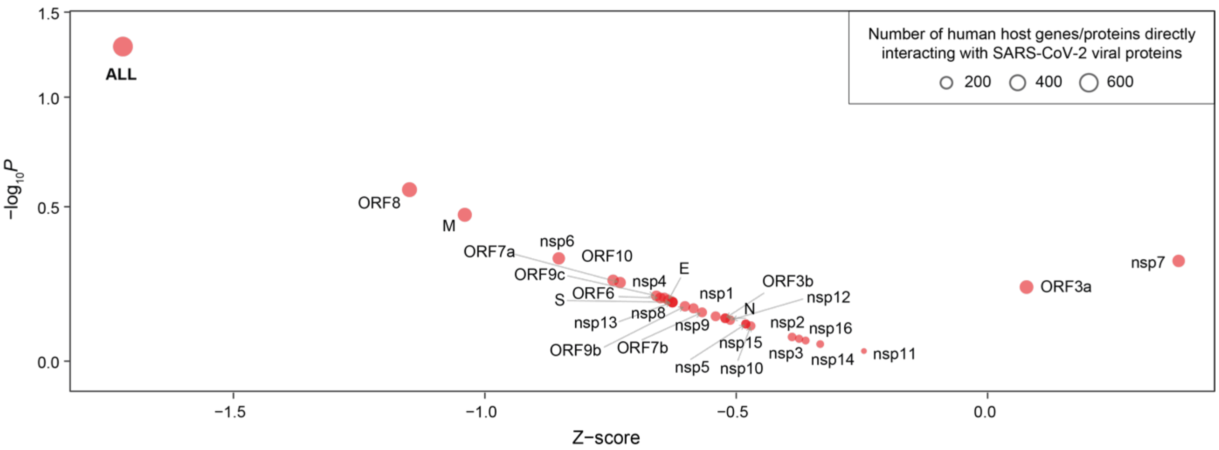


**Supplementary Figure 3: Scatter plot illustrating enrichments of top 1% prioritised genes in human proteins directly interacting with SARS-CoV-2 viral proteins.** Results are shown for each of 29 viral proteins, along with their combinations (i.e., ‘*ALL*’ denoting all human proteins that directly interact with any viral proteins).

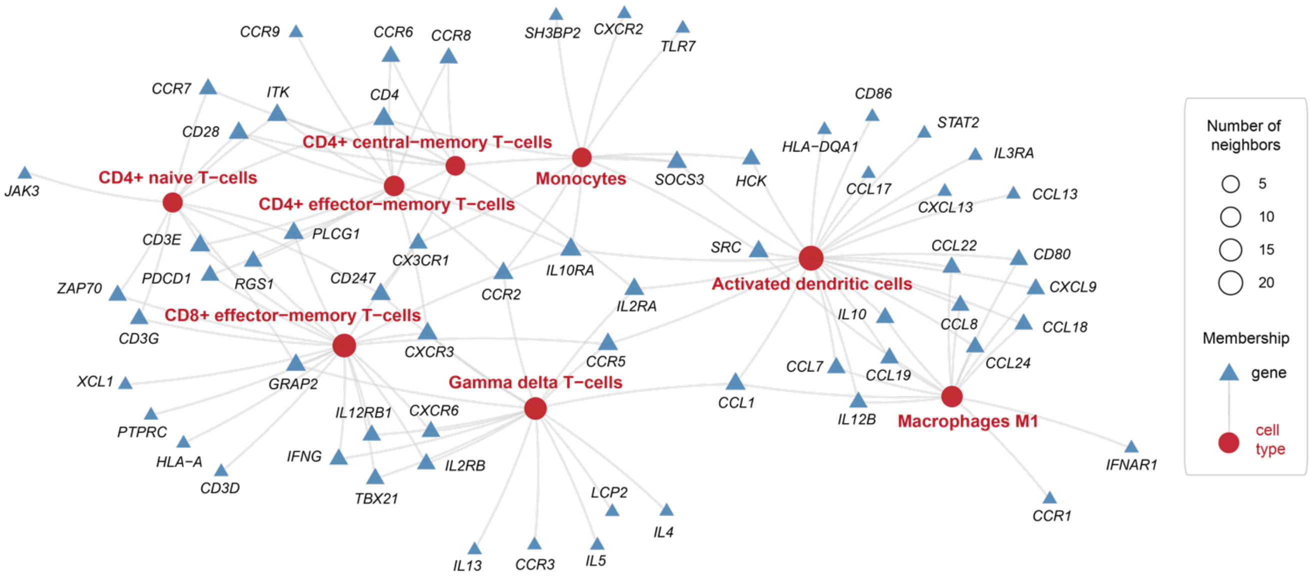


**Supplementary Figure 4: Cell type enrichments for leading target genes.** A bipartite graph connects enriched cell types (represented by red circles) with their respective member genes (indicated by blue triangles).

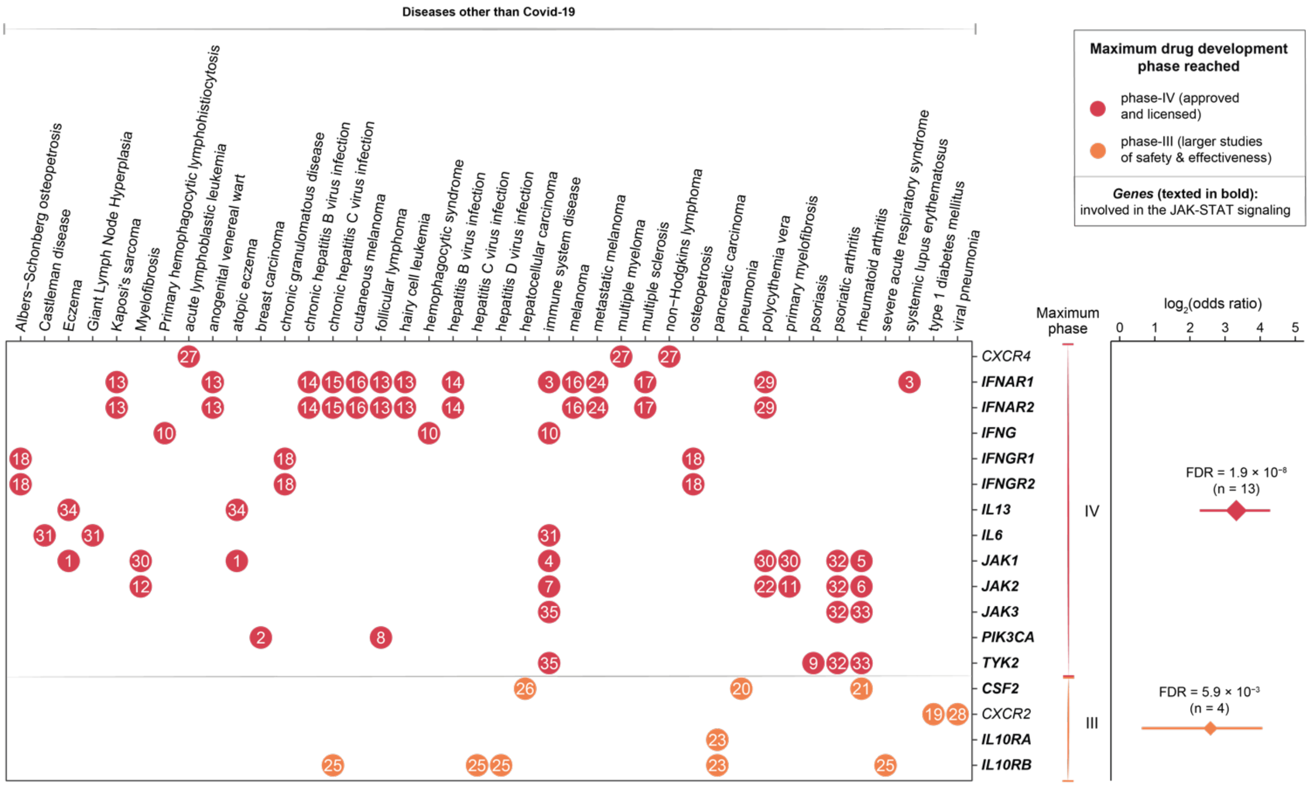


| | Drugs [mechanisms of action] (maximum development phase reached) |
|---|---|
| 1 | abrocitinib [Tyrosine−protein kinase JAK1 inhibitor] (phase−IV) |
| 2 | alpelisib [PI3−kinase p110−alpha subunit inhibitor] (phase−IV) |
| 3 | anifrolumab [Interferon−alpha/beta receptor alpha chain antagonist] (phase−IV) |
| 4 | baricitinib [Tyrosine−protein kinase JAK1 inhibitor] (phase−IV)<br>filgotinib [Tyrosine−protein kinase JAK1 inhibitor] (phase−IV)<br>upadacitinib [Janus Kinase (JAK) inhibitor] (phase−IV) |
| 5 | baricitinib [Tyrosine−protein kinase JAK1 inhibitor] (phase−IV)<br>tofacitinib citrate [Janus Kinase (JAK) inhibitor] (phase−IV)<br>upadacitinib [Janus Kinase (JAK) inhibitor] (phase−IV) |
| 6 | baricitinib [Tyrosine−protein kinase JAK2 inhibitor] (phase−IV)<br>tofacitinib citrate [Janus Kinase (JAK) inhibitor] (phase−IV)<br>upadacitinib [Janus Kinase (JAK) inhibitor] (phase−IV) |
| 7 | baricitinib [Tyrosine−protein kinase JAK2 inhibitor] (phase−IV)<br>upadacitinib [Janus Kinase (JAK) inhibitor] (phase−IV) |
| 8 | copanlisib hydrochloride [PI3−kinase p110−alpha subunit inhibitor] (phase−IV) |
| 9 | deucravacitinib [Tyrosine−protein kinase TYK2 negative allosteric modulator] (phase−IV) |
| 10 | emapalumab [Interferon gamma inhibitor] (phase−IV) |
| 11 | fedratinib hydrochloride [Tyrosine−protein kinase JAK2 inhibitor] (phase−IV)<br>pacritinib citrate [Tyrosine−protein kinase JAK2 inhibitor] (phase−IV)<br>ruxolitinib phosphate [Tyrosine−protein kinase JAK2 inhibitor] (phase−IV) |
| 12 | fedratinib hydrochloride [Tyrosine−protein kinase JAK2 inhibitor] (phase−IV)<br>ruxolitinib phosphate [Tyrosine−protein kinase JAK2 inhibitor] (phase−IV) |
| 13 | interferon alfa−2b [Interferon alpha/beta receptor agonist] (phase−IV) |
| 14 | interferon alfa−2b [Interferon alpha/beta receptor agonist] (phase−IV)<br>peginterferon alfa−2a [Interferon alpha/beta receptor agonist] (phase−IV) |
| 15 | interferon alfa−2b [Interferon alpha/beta receptor agonist] (phase−IV)<br>peginterferon alfa−2a [Interferon alpha/beta receptor agonist] (phase−IV)<br>peginterferon alfa−2b [Interferon alpha/beta receptor agonist] (phase−IV) |
| 16 | interferon alfa−2b [Interferon alpha/beta receptor agonist] (phase−IV)<br>peginterferon alfa−2b [Interferon alpha/beta receptor agonist] (phase−IV) |
| 17 | interferon beta−1a [Interferon alpha/beta receptor agonist] (phase−IV)<br>interferon beta−1b [Interferon alpha/beta receptor agonist] (phase−IV)<br>peginterferon beta−1a [Interferon alpha/beta receptor agonist] (phase−IV) |
| 18 | interferon gamma−1b [Interferon gamma receptor agonist] (phase−IV) |
| 19 | ladarixin [Interleukin−8 receptor B modulator] (phase−III)<br>reparixin [Interleukin−8 receptor B modulator] (phase−III) |
| 20 | lenzilumab [Granulocyte−macrophage colony−stimulating factor inhibitor] (phase−III) |
| 21 | otilimab [Granulocyte−macrophage colony−stimulating factor inhibitor] (phase−III) |
| 22 | pacritinib citrate [Tyrosine−protein kinase JAK2 inhibitor] (phase−IV)<br>ruxolitinib phosphate [Tyrosine−protein kinase JAK2 inhibitor] (phase−IV) |
| 23 | pegilodecakin [IL−10 receptor agonist] (phase−III) |
| 24 | peginterferon alfa−2b [Interferon alpha/beta receptor agonist] (phase−IV) |
| 25 | peginterferon lambda−1a [Interferon lambda receptor agonist] (phase−III) |
| 26 | pexastimogene devacirepvec [Granulocyte−macrophage colony−stimulating factor vaccine antigen] (phase−III) |
| 27 | plerixafor [C−X−C chemokine receptor type 4 partial agonist] (phase−IV) |
| 28 | reparixin [Interleukin−8 receptor B modulator] (phase−III) |
| 29 | ropeginterferon alfa−2b [Interferon alpha/beta receptor positive modulator] (phase−IV) |
| 30 | ruxolitinib phosphate [Tyrosine−protein kinase JAK1 inhibitor] (phase−IV) |
| 31 | siltuximab [Interleukin−6 inhibitor] (phase−IV) |
| 32 | tofacitinib citrate [Janus Kinase (JAK) inhibitor] (phase−IV) |
| 33 | tofacitinib citrate [Janus Kinase (JAK) inhibitor] (phase−IV)<br>upadacitinib [Janus Kinase (JAK) inhibitor] (phase−IV) |
| 34 | tralokinumab [Interleukin−13 inhibitor] (phase−IV) |
| 35 | upadacitinib [Janus Kinase (JAK) inhibitor] (phase−IV) |

**Supplementary Figure 5: Dot plot depicting 17 crosstalk genes (y-axis) currently targeted by approved (phase-IV) and phase-III drugs in diseases other than Covid-19 (x-axis).** Dots are color-coded based on the maximum drug development phase and referenced in integers. Bottom panel: information on referenced drugs and their mechanisms of action. Top-right panel: forest plots of approved or phase-III drug targets enriched in crosstalk genes. The significance level (FDR), odds ratio, and its 95% CI (represented by lines) were calculated using one-sided Fisher's exact test.
